# Validation of IASLC 9th edition TNM classification for lung cancer: focus on N descriptor

**DOI:** 10.1186/s12885-024-13139-z

**Published:** 2024-11-27

**Authors:** Joung Woo Son, Joonseok Lee, Jae Hyun Jeon, Sukki Cho, Woohyun Jung, Beatrice Chia-Hui Shih, Kwhanmien Kim, Sanghoon Jheon

**Affiliations:** Department of Thoracic and Cardiovascular Surgery, Seoul National University Bundang Hospital, Seoul National University College of Medicine, 82, Gumi-ro 173 Beon-gil, Bundang-gu, Seongnam-si, 13620 Gyeonggi-do Republic of Korea

**Keywords:** Non-small cell lung cancer, TNM classification, Nodal stage, External validation, Prognostic validity

## Abstract

**Background:**

We externally validated the proposed 9th edition of the TNM staging classification with our institution’s prospectively collected data and compared it to the 8th edition for overall survival (OS) and freedom from recurrence (FFR).

**Methods:**

A retrospective analysis was conducted of 4029 cases of stage I-III non-small cell lung cancer that underwent surgical treatment from January 2004 to December 2020. Survival was compared using Kaplan-Meier curves and multivariable Cox regression analysis. The concordance index (C-index), Alkaike information criterion (AIC), and R^2^ were used to assess the discriminatory ability.

**Results:**

In the 9th edition, the N2 category (*n* = 352) was subdivided into N2a (*n* = 256, 72.7%) and N2b (*n* = 96, 27.3%). The TNM stage changes were as follows: (1) IIB to IIA, 151 cases (26.0%); (2) IIIA to IIB, 52 cases (11.5%); (3) IIIB to IIIA, 57 cases (61.3%); (4) IIIA to IIIB, 56 cases (12.4%). The survival curves of the proposed 9th edition demonstrated similar patterns to those of the 8th edition, but with a greater discriminative ability for OS and FFR. Subdividing N2 into N2a and N2b refined prognosis prediction. The C-index, AIC, and R2 demonstrated improved values in the proposed 9th edition compared to the 8th edition.

**Conclusions:**

The proposed 9th edition of the TNM staging classification for lung cancer showed favorable prognostic validity and better discrimination ability than the 8th edition.

## Background

Lung cancer is the most common cancer worldwide and ranks as the leading cause of cancer-related death globally [1]. Therefore, accurate staging of lung cancer is crucial for predicting outcomes—most notably, survival and recurrence—as well as for determining appropriate treatments for patients. The International Association for the Study of Lung Cancer (IASLC) has established an international, large-scale, multi-center database and has developed a standardized tumor-node-metastasis (TNM) staging classification according to the anatomic extent of a malignancy. The most recently published 8th edition of the TNM staging classification contained significant revisions of the T descriptor but no changes of the N descriptors, which have historically been categorized based on the anatomic location since the 4th edition [2, 3]. 

To propose the 9th edition of the cancer staging system, the IASLC collected data from a newly established database comprising 124,581 cases gathered from 25 countries [4]. After several stages of data processing, a final total of 87,043 cases were used for the analysis. The IASLC recommended retaining the previous N0, N1, N2, and N3 descriptors, while introducing a new subdivision within the N2 category to differentiate between single-station and multi-station involvement. Additionally, modifications to the TNM staging classification were proposed to align with the changes in the sub-descriptors of the N2 group. However, outcomes based on TNM staging may vary depending on the source of the database, due to its inherent heterogeneity. As with previous editions, the new guidelines should undergo multiple external validations [5–11]. Furthermore, the current database is focused solely on survival and does not include information regarding tumor recurrence.

Therefore, we performed external validation of the proposed 9th edition of the TNM staging classification and N descriptors by using prospectively collected data from our institution. Specifically, we compared the discrimination ability of the 8th and proposed 9th editions in terms of overall survival (OS) and freedom from recurrence (FFR). We also conducted an explorative analysis according to the skip metastasis of single-station N2. On the basis of our analysis, we made suggestions for the next TNM classification.

## Methods

### Patient selection

We conducted a retrospective study on patients diagnosed with non-small cell lung cancer (NSCLC) who underwent surgery at Seoul National University Bundang Hospital from January 2004 to December 2020. These patients underwent complete curative resection (R0), including systemic hilar or mediastinal lymphadenectomy, and were diagnosed with pathologic stage I – III NSCLC (*n* = 4565). The following patients were excluded from the study: those who received neoadjuvant treatment before surgery (*n* = 275), those with a history of prior pulmonary resection surgery for lung cancer (*n* = 119), those who underwent wedge resection (*n* = 107), those with incomplete medical records (*n* = 78), those with N3 disease (*n* = 8), and those who experienced 30-day mortality (*n* = 19). As a result, a total of 4029 patients were included in the study, which was approved by the SNUBH Institutional Review Board (B-2401-875-102, 2023 − 1218). The requirement for informed consent was waived due to the retrospective nature of the study.

### Clinical and pathological data collection

We collected information through a prospective lung cancer database, which included baseline characteristics, such as age, sex, smoking history, and Eastern Cooperative Oncology Group performance status. We also investigated tumor size, pathologic TNM stage, and histology, as well as data on the extent of surgery, numbers of harvested lymph nodes, the administration of adjuvant chemotherapy/radiotherapy, the duration of follow-up, and the occurrence and timing of death and recurrence.

Based on postoperative pathologic findings, we classified patients according to both the 8th and proposed 9th editions of the TNM staging system, which was presented at 2023 World Conference on Lung Cancer [12]. The classification process was carried out through discussions between two experienced thoracic surgeons (J Lee and J-W Son). The T stage categories remained consistent between the two editions [13]. However, in the proposed 9th edition, nodal stage classification differed from the previous N2 category (metastasis in ipsilateral mediastinal and/or subcarinal lymph node(s)), as it was based on the number of N2 stations, regardless of N1 metastasis [14]. This category included single-station N2 metastasis (N2a) and multiple-station N2 metastasis (N2b).

### Surgical procedure including lymphadenectomy

Complete resection of lung cancer and systemic hilar or mediastinal lymphadenectomy were routinely performed by video-assisted thoracic surgery or open thoracotomy. The systematic lymph node dissection was determined based on the recommendations of the Japan Lung Cancer Society [15] and the European Society of Thoracic Surgeons [16]: (1) resection of at least three LNs or three stations from the hilar and intrapulmonary nodes, (2) resection of at least three LNs or three stations from the mediastinal nodes, and (3) resection of at least six LNs or six stations in total.

### Postoperative follow-up strategy

Patients underwent contrast-enhanced chest computed tomography (CT) scans every 3 to 6 months for the first 2 years, followed by scans every 6 months for up to 5 years. Lesions suspected of recurrence were subjected to histological confirmation when clinically feasible. If obtaining sufficient tissue samples was not possible, positron emission tomography CT scans or contrast-enhanced chest CT scans were conducted at intervals of 3 to 6 months for at least 1 year. Recurrence status was clinically assessed through multidisciplinary consultations. The last follow-up date, and when applicable, the dates of death or recurrence, were recorded.

### Study end-point

The primary end-point was the estimated 5-year OS and 5-year FFR according to the 8th and proposed 9th editions of the TNM staging classification. OS was defined as the time from the date of surgery to the date of death or the last follow-up. FFR was determined from the date of surgery to the date of recurrence or the last follow-up. Additionally, exploratory analyses subdivided the N2a group based on the presence of skip metastasis to a single N2 station. The N2a group was categorized into two subgroups: single station N2 with skip metastasis, indicating no N1 involvement (N2a1), and single station N2 without skip metastasis, indicating concurrent N1 involvement (N2a2).

### Statistical analysis

Continuous variables were expressed as the median with the interquartile range (IQR; first to third quartiles), and categorical variables were presented as the total number with the corresponding percentage. The survival curves for OS and FFR were estimated using the Kaplan-Meier method and compared using the log-rank test. Multivariable Cox regression analyses were conducted to adjust for clinical and pathological factors, including age, sex, smoking history, performance status, histologic type, extent of surgery, and adjuvant chemotherapy. Hazard ratios (HRs) and 95% confidence intervals (CIs) were calculated between adjacent stage groups. The prognostic values of the two final multivariate models were assessed using the Akaike information criterion (AIC), for which a lower value indicates a better prognostic prediction model [17]. The R [2] measure and Harrell’s concordance index (C-index) were used to determine the discriminatory power of the 8th and proposed 9th editions of the TNM staging classification. Higher R [2] and C-index values can be regarded as indicating better discrimination ability [18, 19]. All *P*-values less than 0.05 were considered statistically significant. The statistical analysis was conducted with SPSS 22.0 for Windows (IBM Corporation, Armonk, NY, USA) and R statistical software (version 4.1.0; R Foundation for Statistical Computing, Vienna, Austria).

## Results

### Patient characteristics

**Fig. 1:**
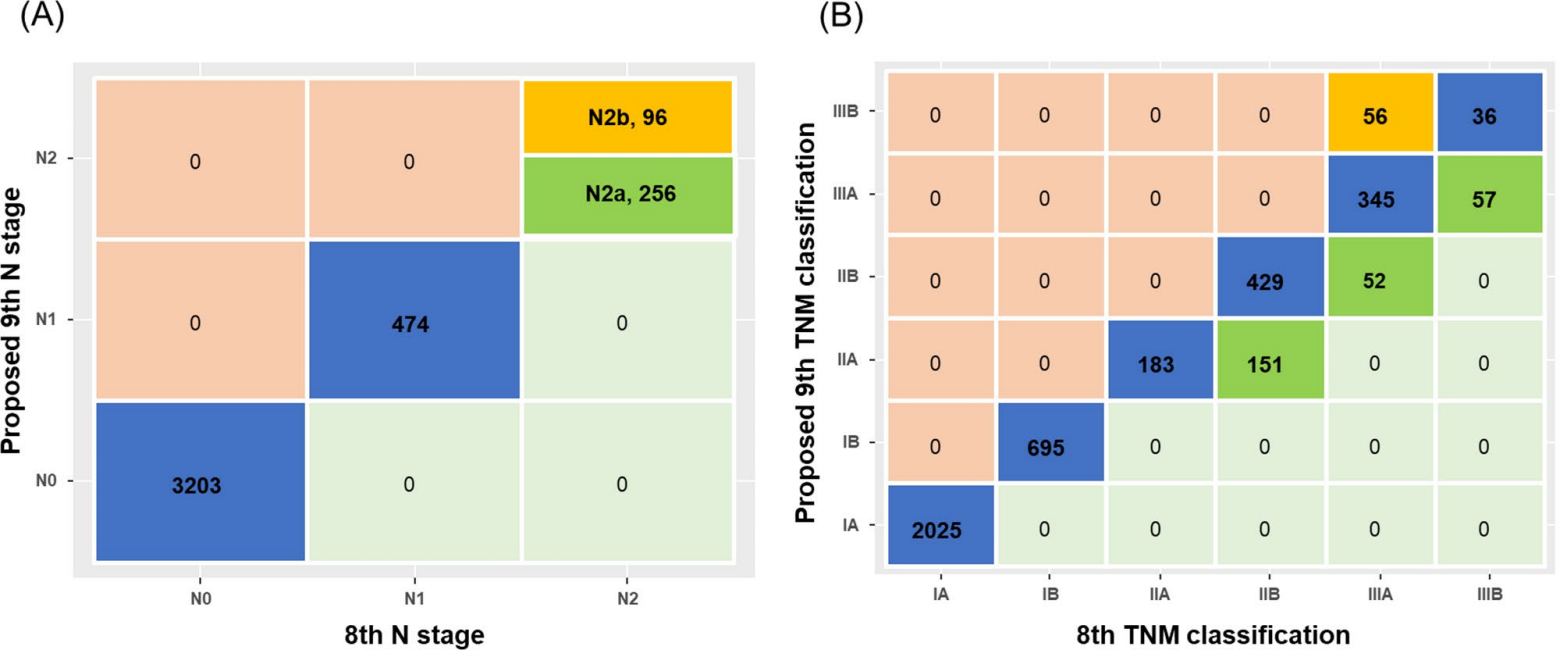
Cross table plots of 8th and proposed 9th edition TNM staging classification (**A**) and N staging classification (**B**)

The clinical and pathological characteristics of the included patients are summarized in Table 1. The median age was 66.0 years (IQR: 58.0 to 72.0 years). The most frequently performed surgery was lobectomy (*n* = 3553, 88.2%) and the median number of harvested lymph node was 17.0 (IQR: 11.0 to 24.0). Adenocarcinoma (*n* = 3017, 74.9%) was the most common histologic cell type, and 1225 patients (30.4%) received adjuvant chemotherapy. The median follow-up period was 6.1 years (IQR: 4.0 to 9.7 years). The TNM staging classification was categorized according to the 8th and proposed 9th editions and summarized in Fig. 1. The patients classified as N2 in the 8th edition were further divided into N2a (256 patients, 72.7%) and N2b (96 patients, 27.3%) according to the proposed 9th edition (Fig. 1A). Some patients experienced changes in TNM staging when the 9th edition was used, as follows: 151 patients (26.0%) were down-staged from stage IIB to IIA, 56 patients (12.4%) were up-staged from IIIA to IIIB, 52 patients (11.5%) were down-staged from IIIA to IIB, and 57 patients (61.3%) were down-staged from IIIB to IIIA (Fig. 1B).

**Table 1:** Clinical and pathological characteristics of included patients (*N* = 4029)

Variables	Included patients: *N* = 4029 ^a^
Age, years	66.0 (58.0–72.0)
Gender, male	2324 (57.7)
Smoking history, yes	2182 (54.2)
Performance status, >=2	624 (15.5)
Tumor size, cm	2.5 (1.7–3.5)
Pathologic T stage, 8th edition	
- T1	2242 (55.6)
- T2	1293 (32.1)
- T3, T4	351 (8.7), 143 (3.5)
Numbers of total lymph nodes	17.0 (11.0–24.0)
Numbers of N1 lymph nodes	8.0 (5.0–11.0)
Numbers of N2 lymph nodes	9.0 (5.0–15.0)
Pathologic N stage, 8th edition	
- N0	3203 (79.5)
- N1, N2	474 (11.8), 352 (8.7)
Visceral pleural invasion, yes	1003 (24.9)
Vascular invasion, yes	729 (18.1)
Lymphatic invasion, yes	1331 (33.1)
Histology	
- Adenocarcinoma	3017 (74.9)
- Squamous cell carcinoma, Others	755 (18.7), 257 (6.4)
Extent of surgery	
- Segmentectomy	318 (7.9)
- Lobectomy	3553 (88.2)
- Bi-lobectomy, Pneumonectomy	92 (2.3), 66 (1.6)
Adjuvant radiotherapy, yes	179 (4.4)
Adjuvant chemotherapy, yes	1225 (30.4)
Follow-up duration, years	6.1 (4.1–10.0)

^a^ Median (1st to 3rd interquartile range), number (%)

### Survival analyses according to the 8th and proposed 9th editions of the TNM staging classifications

**Fig. 2:**
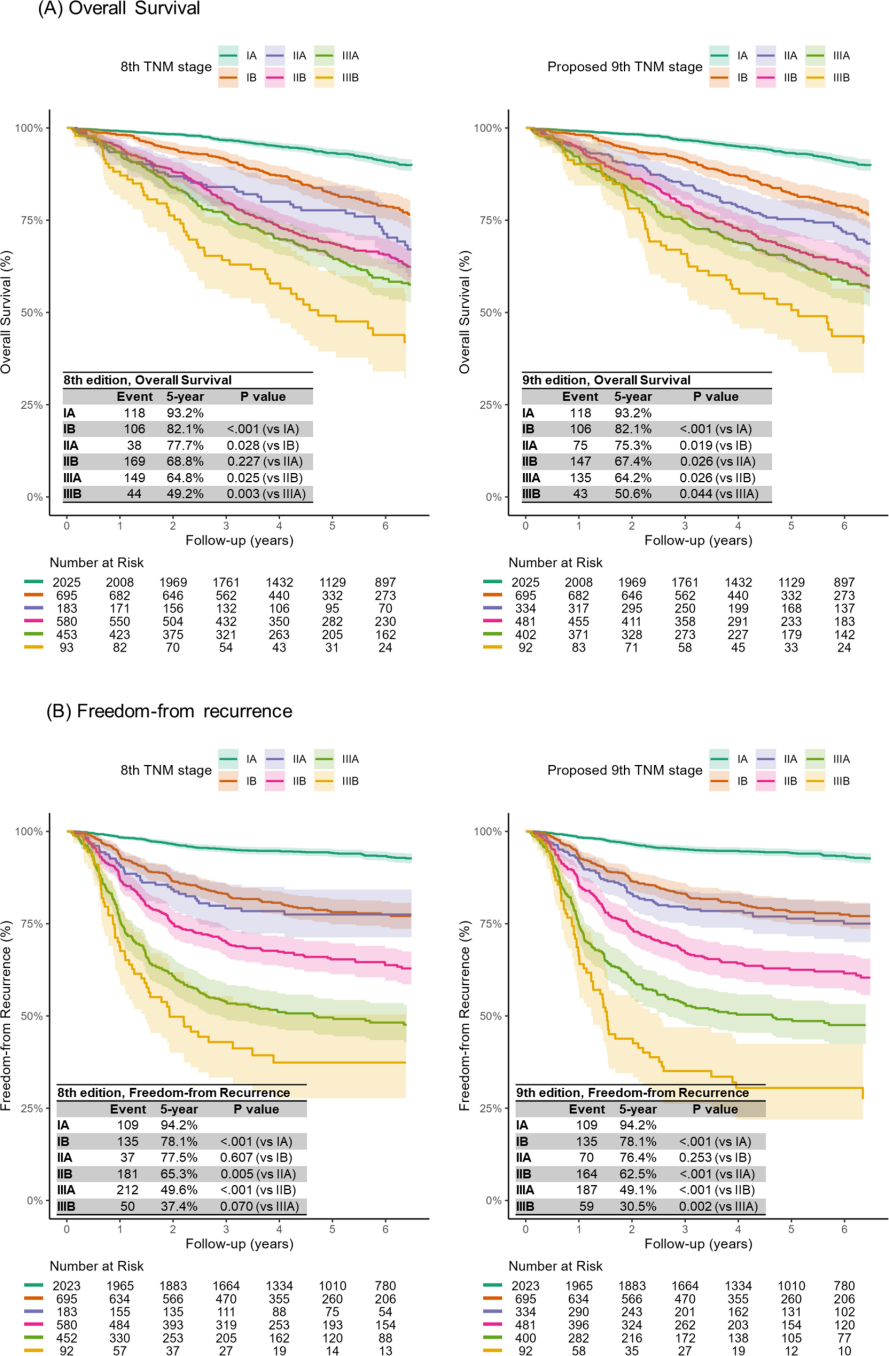
Survival curves of overall survival (**A**) and freedom-from recurrence (**B**) based on the 8th and proposed 9th edition of the TNM staging classification

**Fig. 3:**
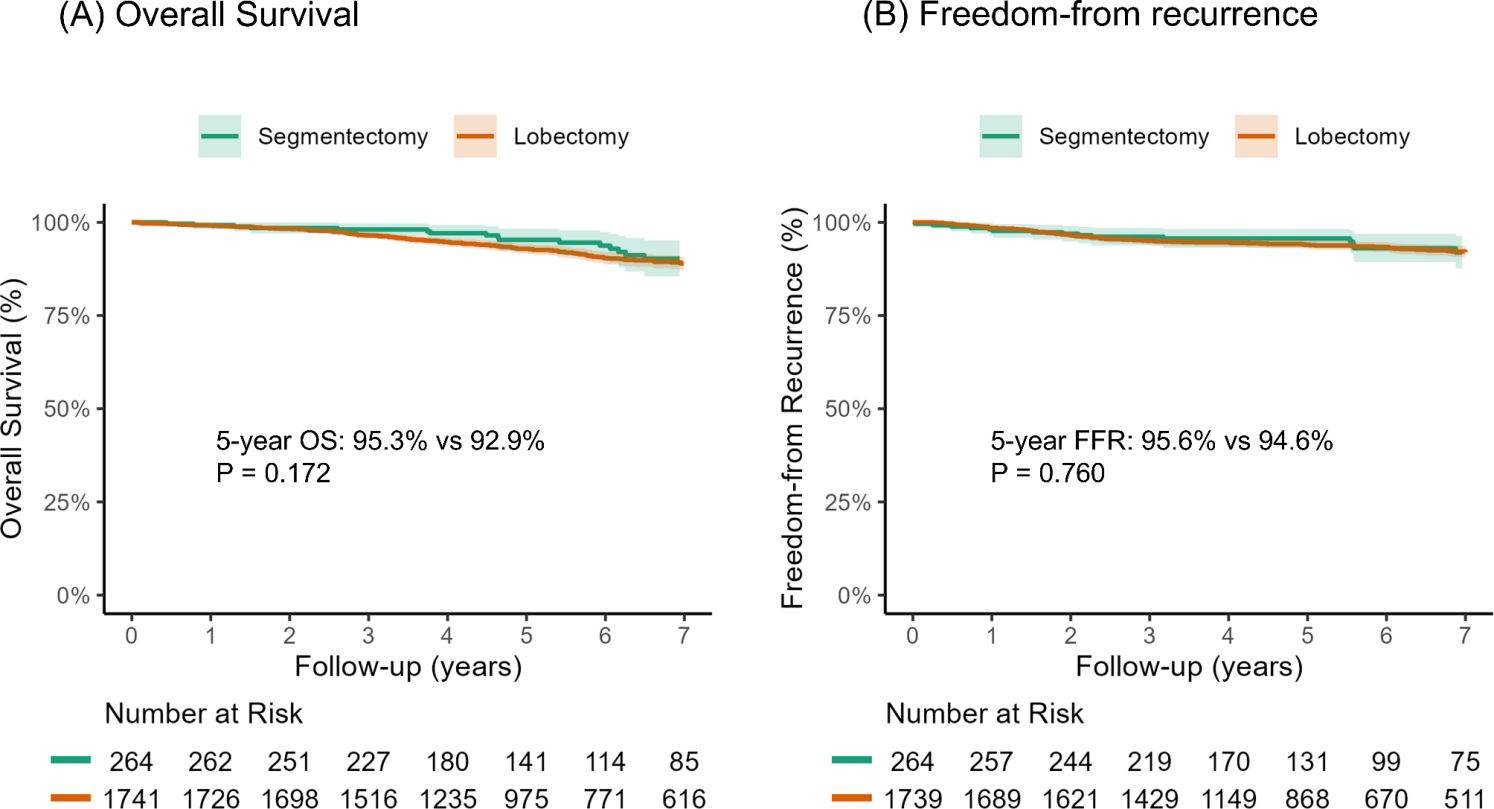
Survival curves of overall survival (**A**) and freedom-from recurrence (**B**) between the segmentectomy and the lobectomy group in patients diagnosed with pathologic stage IA

In Fig. 2, we present the OS and FFR for bothdkdn the 8th and proposed 9th editions of the TNM staging classification. In the 8th edition, the survival curves of OS and FFR demonstrated a stepwise decline from stage IA to IIIB. However, the OS curves of stage IIA and IIB and the FFR curves of stage IB and IIA were not significantly different (*P* = 0.227 and *P* = 0.607, respectively). According to the proposed 9th edition, the survival curves of OS and FFR also displayed a stepwise degradation, and there were clearer differences in the OS curves of stage IIA and IIB (*P* = 0.026) and FFR curves of stage IB and IIA (*P* = 0.253). The exploratory analysis compared survival outcomes between segmentectomy and lobectomy in patients with pathologic stage IA (Fig. 3). The 5-year OS and FFR were 95.3% (95% CI, 92.5–98.3%) and 95.6% (95% CI, 93.2–98.2%) in the segmentectomy group, which showed comparable outcomes to the lobectomy group (5-year OS, 92.9% [95% CI, 91.6–94.2%], *P* = 0.172; 5-year FFR, 94.6% [95% CI, 92.8–95.2%], *P* = 0.760).

The multivariable Cox regression analysis consistently revealed HR values above 1.0 when comparing adjacent stages for both OS and FFR (Table 2). However, a significant difference was not observed between stages IB and IIA in both editions for OS and FFR (8th edition, *P* = 0.224 for OS, *P* = 0.601 for FFR; proposed 9th edition, *P* = 0.056 for OS, *P* = 0.358 for FFR). The AIC, R [2], and C-index demonstrated the superiority of the proposed 9th edition of TNM staging classification compared to the 8th edition, with a lower AIC value (OS: 13670 vs. 13660, FFR: 11904 vs.11878), a higher R [2] value (OS: 0.603 vs. 0.608, FFR: 0.590 vs. 0.603), and a higher C-index (OS: 0.778 vs. 0.779, FFR: 0.765 vs. 0.769).

**Table 2:** Multivariable Cox proportional hazard model for 8th and proposed 9th TNM staging classification

	Overall Survival					Freedom-from Recurrence			
	8th TNM		Proposed 9th TNM			8th TNM		Proposed 9th TNM	
	HR (95% CI)	*P*	HR (95% CI)	*P*		HR (95% CI)	*P*	HR (95% CI)	*P*
IB (vs. IA)	2.05 (1.67–2.53)	< 0.001	2.03 (1.65–2.50)	< 0.001		3.32 (2.59–4.25)	< 0.001	3.30 (2.58–4.22)	< 0.001
IIA (vs. IB)	1.20 (0.89–1.62)	0.224	1.27 (0.99–1.63)	0.056		1.10 (0.77–1.57)	0.601	1.14 (0.86–1.51)	0.358
IIB (vs. IIA)	1.39 (1.03–1.86)	0.029	1.37 (1.08–1.74)	0.009		1.58 (1.11–2.24)	0.010	1.64 (1.25–2.15)	< 0.001
IIIA (vs. IIB)	1.32 (1.09–1.59)	0.005	1.25 (1.03–1.53)	0.027		1.64 (1.35–1.99)	< 0.001	1.51 (1.23–1.86)	< 0.001
IIIB (vs. IIIA)	1.54 (1.15–2.08)	0.004	1.84 (1.35–2.51)	< 0.001		1.39 (1.02–1.89)	0.037	1.79 (1.33–2.39)	< 0.001
AIC, R2, Harrell’s C-index	13,670, 0.603, 0.778		13,660, 0.608, 0.779			11,904, 0.590, 0.765		11,878, 0.603, 0.769	

AIC: Akaike information criterion; CI: confidence interval: C-index: concordance index; HR: hazard ratio; TNM, tumor, node, and metastasis

### Survival analyses according to the 8th and proposed 9th N staging classifications

**Fig. 4:**
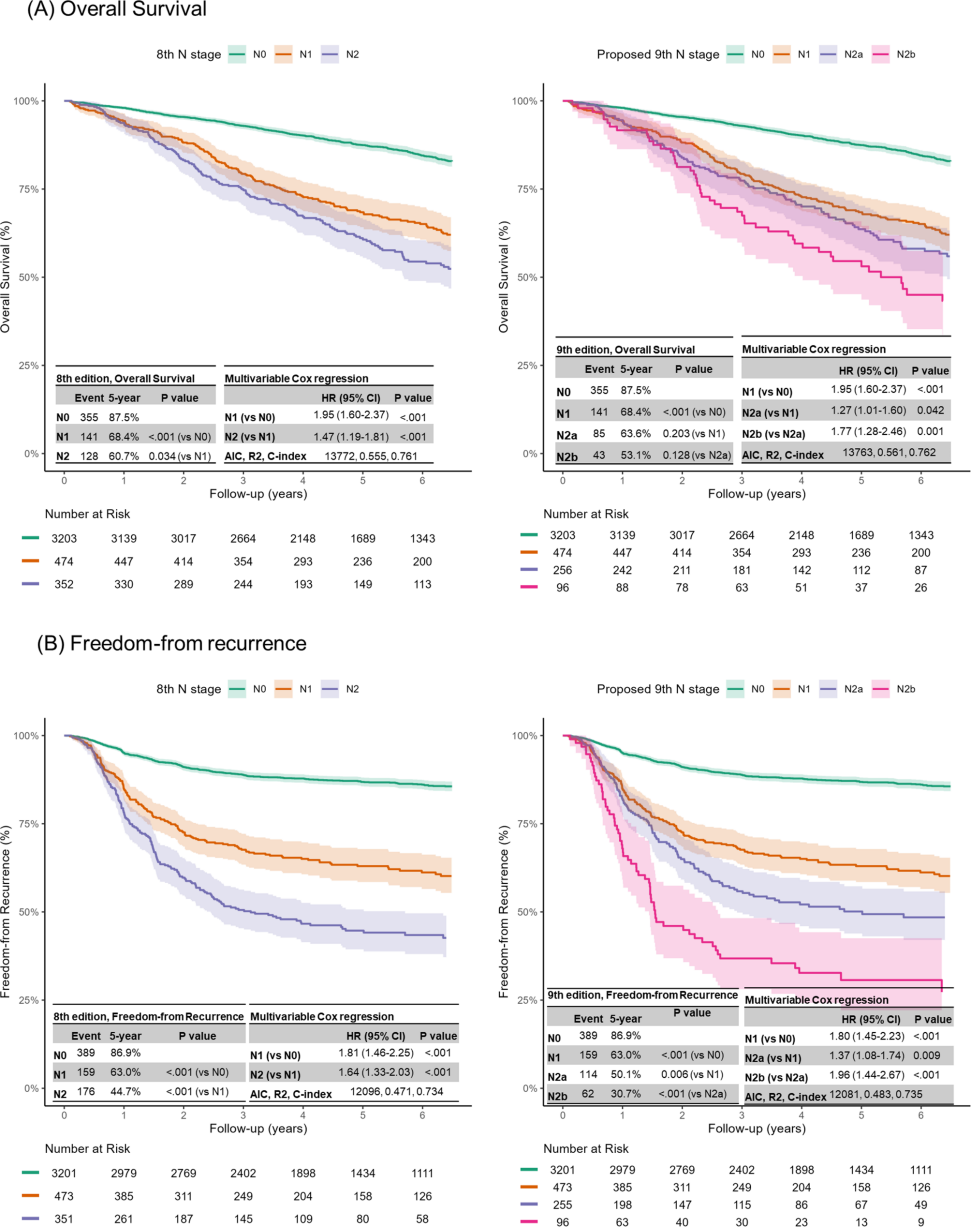
Survival curves of overall survival (**A**) and freedom-from recurrence (**B**) based on the 8th and proposed 9th edition of the N staging classification

Figure 4A shows sequential deteriorations in OS in both the 8th and proposed 9th editions of the N stage. According to the proposed 9th edition, the OS curves between the N1 group and N2a group were not significantly different (*P* = 0.203). However, according to multivariable Cox proportional hazard analysis, all HRs between adjacent N staging groups were higher than 1.0, indicating a gradual decline in the prognosis according to the proposed N staging groups (Fig. 3A). The AIC, R [2], and C-index values consistently demonstrated better prediction accuracy and discriminatory ability for the proposed 9th edition than for the 8th edition (AIC: 13763 vs. 13772, R [2]: 0.561 vs. 0.555, C-index: 0.762 vs. 0.761).

When applying the 9th edition N staging groups for FFR, the differences across groups were very clear (Fig. 4B). The AIC, R [2], and C-index values consistently demonstrated better prediction accuracy and discriminatory ability for the proposed 9th edition than for the 8th edition (AIC: 12081 vs. 12096, R [2]: 0.483 vs. 0.471, C-index: 0.735 vs. 0.734).

### Exploratory analyses according to the subdivision of the N2a group into N2a1 and N2a2

**Fig. 5:**
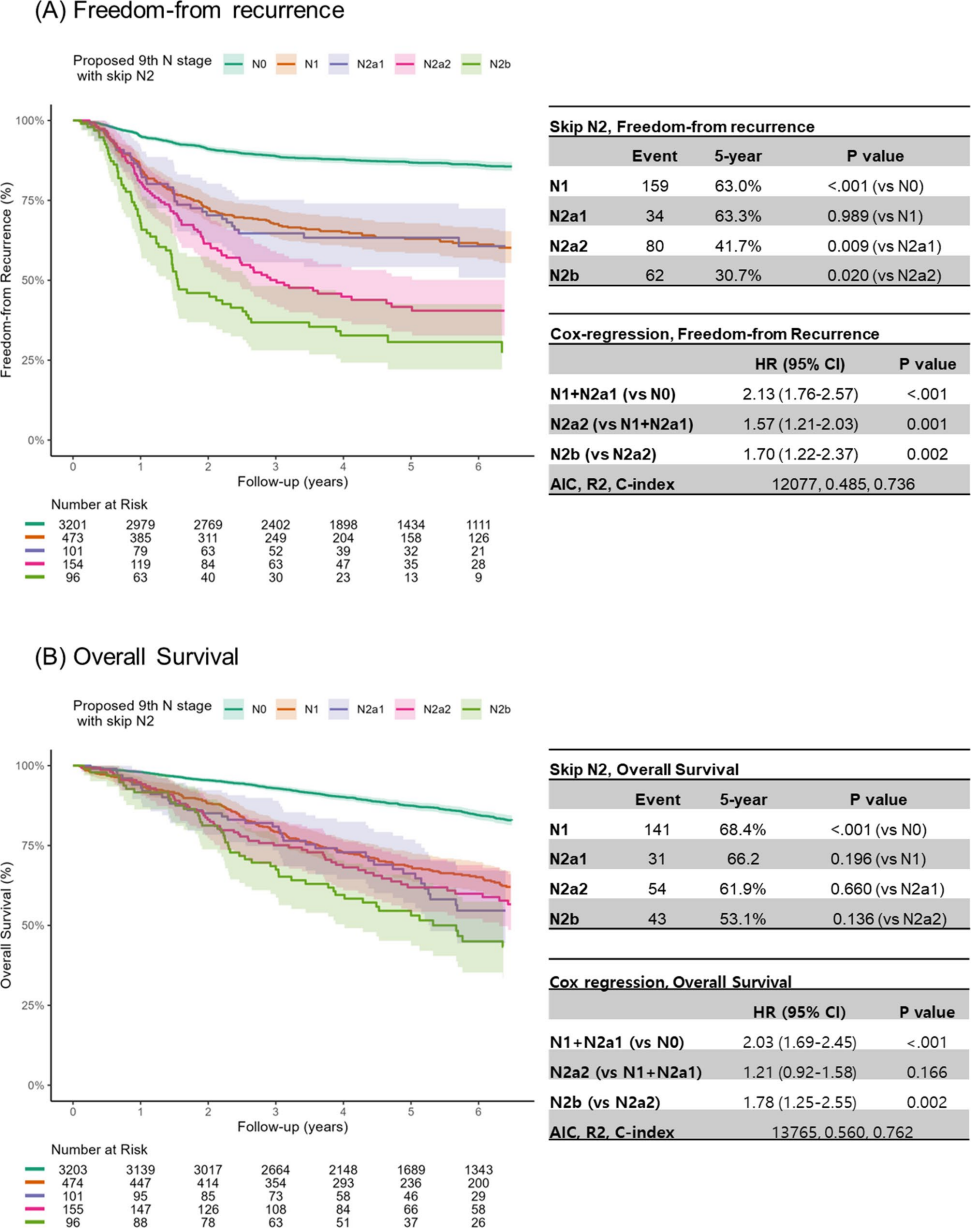
Survival curves of freedom-from recurrence (**A**) and overall survival (**B**) according to subdivision of N2a group as N2a1 and N2a2

We conducted an exploratory analysis according to skip N2 metastasis, which was proposed in the 8th edition but not applied in the 9th edition (Fig. 5). Depending on whether N1 skip metastasis was present, the N2a group (*n* = 256) was divided into N2a1 (*n* = 101) and N2a2 (*n* = 155) groups. There was a significant stepwise worsening of the FFR curves, except for N1 and N2a1 (*P* = 0.989). After grouping N1 and N2a1 into the same category (N1 + N2a1 group), multivariable Cox regression analysis revealed significant HRs between the adjacent groups, with all HR values exceeding 1.0. Using the proposed 9th edition as a reference, AIC, R [2], and C-index values indicated better results after inclusion of skip N2 metastasis (AIC: 12077 vs. 12081, R [2]: 0.485 vs. 0.483, C-index: 0.736 vs. 0.735). However, the gradual increase in recurrence according to skip N2 metastasis was not significantly associated with poorer survival outcomes, indicating some overlapping of the OS curves between N1 and N2a1 (*P* = 0.196), N2a1 and N2a2 (*P* = 0.660), and N2a2 and N2b (*P* = 0.136).

## Discussion

The IASLC Lung Cancer Staging Project, approved by UICC and AJCC, has been building a database since the 1990s and periodically proposing modifications to the TNM staging classification [20–22]. The 8th edition of the classification contained major changes to the T descriptors—namely, only the invasive component was taken into consideration when determining tumor size [23]. At that time, there were proposals to revise the N descriptors to reflect the anatomical location and the number of lymph nodes involved. However, these suggestions were not adopted because the available lymph node data was predominantly sourced from Japan [24]. For the proposed 9th edition, the IASLC has broadened its data sources to include information from Asia, North America, Europe, and other regions. The use of electronic data capture systems in some areas of data collection has also enhanced the accuracy of the IASLC database [4]. Using this database, the IASLC staging committee has suggested dividing the N2 descriptors into two groups, N2a and N2b, based on the number of involved nodal stations.

Our center has been prospectively collecting and managing comprehensive clinical and pathological data for all patients who underwent pulmonary resection surgery for lung cancer since 2004. This prospective database, similar to the IASLC electronic system, includes baseline characteristics, tumor stage, histology, location and number of affected lymph nodes, cause of death, and recurrence. We conducted a validation analysis using this database and demonstrated that the proposed 9th edition has better prognostic and discriminatory power than the 8th edition in both univariable and multivariable analyses. The OS curves for stage IIA and IIB were similar in the 8th edition, while the proposed 9th edition showed better discrimination with no overlap. According to the 8th -edition staging system, stage IIA included tumors of 4 cm to 5 cm with negative lymph nodes (T2bN0), which accounted for fewer patients (4.9%, 180 out of 3711 patients in our study) than other stage groups. However, in the proposed 9th edition, tumors of 3 cm or less with N1 metastasis (T1N1, 140 cases) and N2a metastasis (T1N2a, 51 cases) were reclassified as stage IIA and IIB, respectively. This reclassification might increase the distinguishability between these two groups. In the FFR analysis, the 9th edition allowed better stepwise separation between the subgroups, showing a clearer, albeit insignificant, difference between IB and IIA. This trend remained consistent in the multivariable analysis for FFR and even for the OS analysis. We hypothesize that the outcomes might have been influenced by individual treatment strategies regarding the administration of adjuvant chemotherapy in patients with stage IB cancer [25]. 

The analysis of OS and FFR according to the N descriptor indicated that a more refined prognosis prediction is possible when N2 is subdivided into N2a and N2b. Notably, the survival difference between N1 and N2a was less pronounced than that between N2a and N2b, which suggests that the number of lymph node metastases has a meaningful impact on prognosis [26–33]. Our exploratory analysis also evaluated the importance of quantifying N2 descriptors. We found that patients with skip N2 metastasis had a more favorable prognosis regarding disease recurrence compared to those with non-skip N2 metastasis. However, this trend did not correlate with survival outcomes, and such complex classifications proved challenging to assess in clinical staging.

Our research has certain limitations. First, our data were collected from a single center and included a limited number of patients. Second, although the IASLC data encompass a diverse racial composition, our dataset was exclusively comprised of Asian individuals, which exhibits a clear predominance of adenocarcinomas. Additionally, the pathological reports do not account for the most recent proposed R status, particularly R(un), as our study included patients who underwent complete resection of NSCLC from 2004 to 2020. These factors may introduce potential confounders in evaluating the prognostic and discriminative ability of proposed 9th edition of TNM staging classification. Third, the IASLC data encompass patients who underwent non-surgical treatments, while our dataset was restricted to patients who received surgical treatment, with or without adjuvant therapy, and therefore may not represent the entire patient population.

## Conclusions

The proposed 9th edition of the TNM staging classification demonstrates improved prognostic validity and enhanced discrimination ability compared to the 8th edition. To further refine future staging systems, additional validation studies focusing on the quantification of N descriptors are warranted. These should be conducted through large-scale prospective studies.

## Data Availability

The datasets used and/or analysed during the current study are available from the corresponding author on reasonable request.
